# COVID-19 and spontaneous pneumothorax: a survival analysis

**DOI:** 10.1186/s13019-023-02331-0

**Published:** 2023-07-04

**Authors:** Reza Ershadi, Shahab Rafieian, Mohammadreza Salehi, Hossein Kazemizadeh, Hesam Amini, Marjan Sohrabi, Alireza Samimiat, Yaser Sharafi, Mohadese Dashtkoohi, Matin Vahedi

**Affiliations:** 1 Department of thoracic surgery, Imam Khomeini Hospital Complex, Tehran University of Medical Sciences, Tehran, Iran; 2 Research center of Antibiotic stewardship & Anti-microbial resistance, Infectious diseases department, Imam Khomeini hospital complex, Tehran University of medical sciences, Tehran, Iran; 3 Advanced Thoracic Research Center, Tehran University of Medical Sciences, Tehran, Iran; 4 Department of Infectious Diseases, Imam Khomeini Hospital Complex, Tehran University of Medical Sciences, Tehran, Iran; 5 Department of surgery, Sina Hospital, Tehran University of Medical Sciences, Tehran, Iran; 6 Department of surgery, Sina Hospital, Tehran University pf Medical Sciences, Tehran, Iran; 7 School of Medicine, Tehran University of Medical Sciences, Tehran, Iran

**Keywords:** COVID-19, Pneumothorax, CT scan

## Abstract

**Introduction:**

COVID-19 Patients may be at risk for involving with spontaneous pneumothorax. However, clinical data are lacking in this regard. In this study, we aimed to investigate the demographic, clinical, and radiological characteristics and survival predictors in COVID-19 patients with pneumothorax.

**Methods:**

This is a retrospectivestudy conducted on COVID-19 patients with pneumothorax that had been hospitalized at hospital. l from December 2021 to March 2022. The chest computed tomography (CT) scan of all patients was reviewed by an experienced pulmonologist in search of pulmonary pneumothorax. Survival analysis was conducted to identify the predictors of survival in patients with COVID-19 and pneumothorax.

**Results:**

A total of 67 patients with COVID-19 and pneumothorax were identified. Of these, 40.7% were located in the left lung, 40.7% were in the right lung, and 18.6% were found bilaterally. The most common symptoms in the patient with pneumothorax were dyspnea (65.7%), increased cough severity (53.7%), chest pain (25.4%), and hemoptysis (16.4%). The frequency of pulmonary left and right bullae, pleural effusion, andfungus ball were 22.4%, 22.4%, 22.4%, and 7.5%, respectively. Pneumothorax was managed with chest drain (80.6%), chest drain and surgery (6%), and conservatively (13.4%). The 50-day mortality rate was 52.2% (35 patients). The average survival time for deceased patients was 10.06 (2.17) days.

**Conclusions:**

Our results demonstrated that those with pleural effusion or pulmonary bullae have a lower survival rate. Further studies are required to investigate the incidence and causality relation between COVID-19 and pneumothorax.

## Introduction

The severe acute respiratory syndrome coronavirus 2 (SARS-CoV2) virus was first isolated in Wuhan, China, in December 2019 [1]. Before the development of the reverse transcriptase-polymerase chain reaction (RT-PCR) test, chest computed tomography (CT) was crucial in the diagnosis of COVID-19 [2]. Additionally, chest CT scans aid in the diagnosis of complications and prediction of clinical course and outcome [3, 4]. Chest CT scan sensitivity is 89 to 97% in COVID-19 diagnosis [5–7]. More than 70% of COVID-19 patients with RT-PCR test evidence have been found to show certain chest CT scan characteristic features, such as ground-glass opacities, vascular enlargement, bilateral abnormalities, lower lobe involvement, and posterior inclination [8, 9]. Nonetheless, a number of less common radiological findings have been observed in patients with confirmed COVID-19, including pneumothorax, bullae, pleural effusion, lymphadenopathy, central lesion distribution, and pericardial effusion [4, 10].

In the lung parenchyma, COVID-19 pneumonia may produce cystic lesions that can either disappear or develop into bigger blebs [11, 12]. Patients may be at risk for rupture as a result, which might lead to subsequent spontaneous pneumothorax or mediastinal and subcutaneous emphysema [35]. Primary spontaneous pneumothorax has no recognized etiology, although secondary spontaneous pneumothorax can happen when there is an underlying lung condition [33]. There have been reports of secondary spontaneous pneumothorax as a COVID-19 consequence, with documented frequencies of 1% in hospitalized patients [13], 3% in hospitalized patients with pneumonia [14], 6% in mechanically-ventilated patients [15], and 1% in deceased patients [16]. It is unknown how effectively the damaged lung tissue will mend and re-expand on its own in COVID-19 individuals due to the inadequate understanding of lung histology in these patients [17]. Patients with neutrophilia, extensive lung damage, and a protracted clinical course were more likely to experience pneumothorax [34]. Similar to this, pneumothorax has been recognized as a poor prognostic characteristic of infection associated with the Middle East respiratory syndrome coronavirus (MERS) [18]. It is suggested that prompt diagnosis and management of pneumothorax might decrease the morbidity and mortality in patients with COVID-19 [19, 20]. However, clinical data are lacking in this regard. In this study, we aimed to investigate the demographic, clinical, and radiological characteristics of patients with confirmed COVID-19 and pneumothorax. We intended to see which factors are predictors of survival in patients with COVID-19 and pneumothorax.

## Materials and methods

This was a retrospective clinical study performed on patients with confirmed COVID-19 that had been hospitalized in Imam Khomeini complex hospital from December 2021 to March 2022. Inclusion criteria were: (1) SARS-COV-2 PCR positive from nasopharyngeal swab or respiratory secretion, (2) presence of pneumothorax in chest CT scan of patients with COVID-19 in first admission or During hospitalization. Also, patients with pneumomediastinum in addition to pneumothorax were excluded from study.

Detection of patients with COVID-19 were based on positive nasopharyngeal swab and chest CT involvement. The chest CT scan of all COVID-19 patients In all departments of the hospital were reviewed by an expert pulmonologist in search of pneumothorax, and patients with pneumothorax were included. Demographic, clinical, and therapeutic data were extracted from the hospital information system. Chest CT scans of the patients with pneumothorax were evaluated by a pulmonologist for recording the size and side of pneumothorax, presence of bullae, pleural effusion, and fungus ball and also Determining the severity of lung involvement. The protocol of this study was approved by the ethics committee of the Tehran University of Medical Sciences (R.TUMS.IKHC.REC.1400.340). Our study was conducted in accordance with the Helsinki Declaration of 1964 and its later amendments. Patients were followed for 50 days for live/death outcomes (survival analysis). Our study was retrospective and it was not possible to obtain informed consent.

### Statistical analyses

Data were analyzed using Statistical Package for the Social Sciences 20 (SPSS, Chicago, IL, USA). Data are demonstrated as frequency (percentage) or mean (standard deviation (SD)). Kaplan-Meier analysis was implemented to perform survival. “P values”<0.05 were considered statistically significant.

## Results

### Demographic, clinical, and laboratory characteristics of patients with COVID-19 and pneumothorax

Of 9800 evaluated cases, a total of 67 patients with COVID-19 and pneumothorax were included (0.68%). Mean and SD of age was 49.92 ± 13.22 years (Table 1). Fifty-nine patients (73.1%) were male. The majority of patients had never smoked, but 15 (22.4%) were current smokers and 10 (14.9%) were ex-smokers. In regard to BMI, 26 (48.1%) were overweight and 12 (22.2%) were obese.

**Table 1:** Demographic and clinical characteristics of included patients

Age	Years, Mean (SD)	49.92 (13.22)
	>=60 (%)	16 (25.4%)
Sex [Male, (n, (%))]		49 (73.1%)
Smoking [yes, (n, (%))]	Never	42 (62.7%)
	Current	15 (22.4%)
	Ex-smoker	10 (14.9%)
BMI	Kg/m^2^, Mean (SD)	25.71 (4.10)
	18-24.9 (%)	16 (29.6%)
	25-29.9 (%)	26 (48.1%)
	>=30 (%)	12 (22.2%)
Hospital stay (days, Mean (SD))		19.42 (14.03)
ICU stay	Frequency (%)	32 (47.8%)
	Days, Mean (SD)	16.65 (11.47)
Symptoms of pneumothorax	Increased cough severity	36 (53.7%)
	Dyspnea	44 (65.7%)
	Chest pain	17 (25.4%)
	Hemoptysis	11 (16.4%)
Previous pneumothorax (n, (%))		4 (6%)
comorbidities	HTN (n, (%))	19 (28.4%)
	DM (n, (%))	10 (14.9%)
	HLP (n, (%))	6 (9%)
	Hypothyroidism (n, (%))	7 (10.4%)
Respiratory comorbidities	Asthma (n, (%))	3 (4.5%)
	COPD (n, (%))	1 (1.5%)
	TB (n, (%))	2 (3%)
Severity of COVID-19	Mild (%)	1 (1.5%)
	Moderate (%)	17 (25.4%)
	Severe (%)	48 (71.6%)
Lung with more COVID-19 severity	Left (%)	25 (37.3%)
	Right (%)	39 (58.2%)
	Both (%)	3 (4.5%)
COVID-19 treatments	Remdesivir	53 (79.1%)
	Corticosteroids	54 (80.6%)
	Tocilizumab	31 (46.3%)
Antibiotics (frequency (%))		47 (70.1%)
WBC (Mean (SD)) /ul		15426.86 (18530.72)/ul
Neutrophil (Mean (SD)) %		76.15 (14.38) %
Lymphocytes (Mean (SD)) %		13.63 (8.52) %
Hemoglobin (Mean (SD)) g/dl		12.73 (2.66)g/dl
Platelet (Mean (SD)) /ul		210246.26 (88050.44)/ul
CRP (Mean (SD)) mg/dl		85.16 (48.95) mg/dl

BMI: body mass index; HTN: hypertension; DM: diabetes mellitus; HLP: hyperlipidemia; COPD: chronic obstructive pulmonary disease; TB: tuberculosis; NIV: non-invasive ventilation; ICU: intensive care unit; WBC: white blood cell; CRP: C-reactive protein;

The mean and SD of hospital stay was 19.42 ± 14.03 (1–64) days. Among patients, 32 (47.8%) were treated in ICU and mean ± SD of ICU stay was 16.65 ± 11.47 days. The most common symptoms in patient with pneumothorax were dyspnea (65.7%), increased cough severity (53.7%), chest pain (25.4%), and hemoptysis (16.4%). Only four (6%) patients had a previous history of pneumothorax.

The majority of patients had severe (71.6%) and moderate (25.4%) COVID-19. Our patient population were treated with remdesivir (79.1%), corticosteroids (80.6%), and tocilizumab (46.3%). Moreover, antibiotic therapy was implemented for 70.1% of patients.

The laboratory measurements of patients are also shown in Table 1.

### Radiological and respiratory characteristics of patients with COVID-19 and pneumothorax

Left, right, and bilateral pneumothorax was observed in CT scans of 24 (40.7%), 24 (40.7%), and 11 (18.6%) patients, respectively (Table 2). At the time of pneumothorax diagnosis, 39.3% and 25% of patients needed reserved and simple masks for O2 support, respectively. Moreover, 14.3% were on non-invasive ventilation (NIV) and 21.4% were intubated.

**Table 2:** Respiratory and radiological characteristics of included patients

Side of pneumothorax (frequency (%))	Left	24 (40.7%)
	Right	24 (40.7%)
	Bilateral	11 (18.6%)
Size of pneumothorax (percentage of lung, mean (SD))		21.73 (15.96)
Mode of breathing at the diagnosis of pneumothorax	Mechanical ventilation (frequency (%))	12 (21.4%)
	NIV (frequency (%))	8 (14.3%)
	Reserved mask (frequency (%))	22 (39.3%)
	Simple mask (frequency (%))	14 (25%)
Mechanical ventilation	Frequency (%)	22 (32.8%)
	Days, Mean (SD)	12.62 (10.80)
Right lung Bullae	Existence (frequency (%))	15 (22.4%)
	Number (mean (SD))	2.00 (1.30)
Left lung bullae	Existence (frequency (%))	15 (22.4%)
	Number (mean (SD))	2.46 (2.32)
Pleural effusion (frequency (%))		15 (22.4%)
Fungal ball (frequency (%))		5 (7.5%)

NIV: non-invasive ventilation

Pneumothorax was managed with chest drain (80.6%), chest drain and surgery (6%), and conservatively (13.4%). The duration of chest tube was 11.60 (10.12) days. In terms of survival rate, 23 patients survived and 31 patients died from the patients who were under chest drainage. In addition among the patients who underwent conservative treatment, 5 patients survived and 4 patients died, also of patients who were treated with chest drainage and surgery, 4 patients survived and no patient died, based on statistically evolution there is no significant differences between the two groups (P = 0.075).

### Survival analysis after pneumothorax in patients with COVID-19

**Fig. 1:**
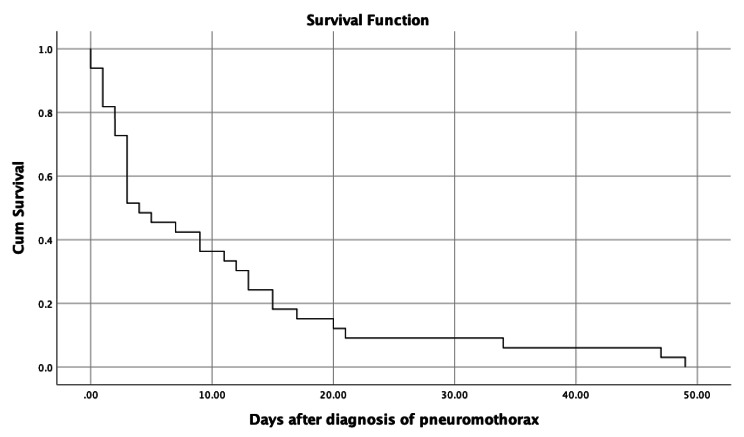
Survival following pneumothorax in patients with COVID-19

The patients were followed for 50 days after diagnosis of pneumothorax. The mortality rate was 52.2% (35 patients) in this period. The average survival time for deceased patients was 10.06 (2.17) days. Figure 1 demonstrates the survival plot of the patients with COVID-19 and pneumothorax (mean, 95% confidence interval (CI) = 10.06 (5.82–14.30)).

### Predictors of survival

We investigated the predictors of survival time in patients with COVID-19 and pneumothorax. For this purpose, we assessed the possible effects of demographic factors (sex, age, smoking, and body mass index (BMI)), respiratory and radiological factors (mechanical ventilation, side of pneumothorax, existence of bullae, and pleural effusion), and treatment factors (remdesivir, corticosteroids, tocilizumab, and intervention for the management of pneumothorax) on the survival of patients.

### Demographic factors

**Fig. 2:**
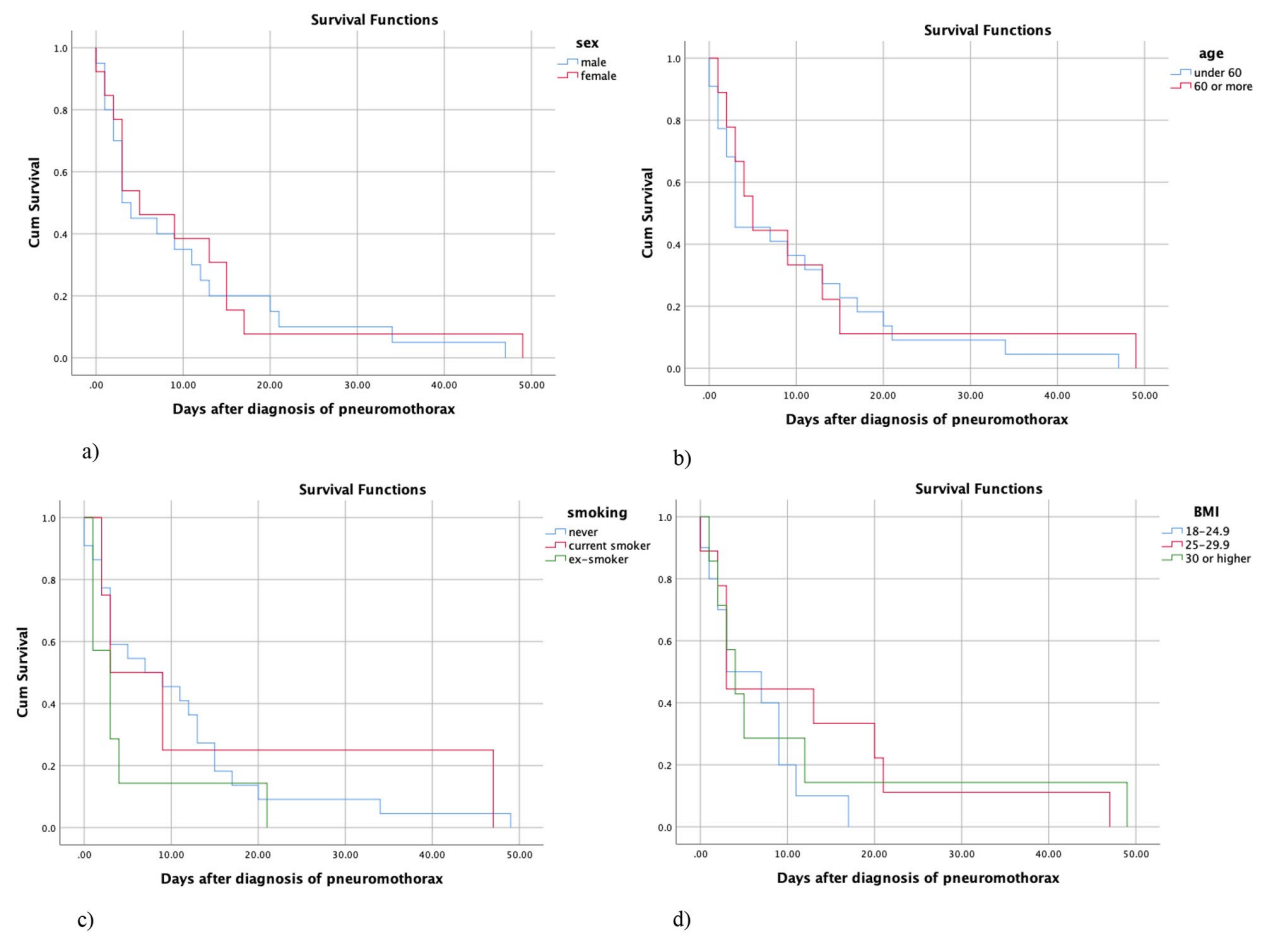
Relationship between sex (**a**), age (**b**), smoking (**c**), BMI (**d**) and survival in patients with COVID-19 and pneumothorax

Our results demonstrated no significant association between the demographic factors, including increased age (p = 0.682), sex (p = 0.637), smoking (p = 0.405), and BMI (p = 0.516) with survival rate (Table 3; Fig. 2).

**Table 3:** Survival analysis and the demographic factors

Variable		Mean	95% CI	Log rank P-value
sex	Male	9.85	4.44–15.25	0.682
	Female	10.38	3.28–17.48	
Age	< 60	9.81	4.76–14.87	0.637
	>=60	11.22	1.42–21.01	
Smoking	Never	10.77	5.82–15.72	0.405
	Current smoker	15.25	0-36.21	
	Ex-smoker	4.85	0-10.20	
BMI	18-24.9	6.20	2.87–9.52	0.516
	25-29.9	12.44	2.49–22.39	
	>=30	10.85	0-23.59	

### Respiratory and radiological factors

**Fig. 3:**
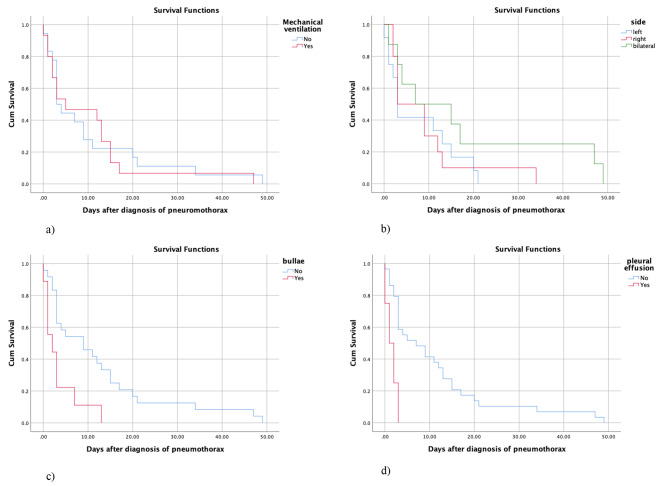
Relationship between mechanical ventilation (**a**), side of pneumothorax (**b**), presence of bullae (**c**), presence of pleural effusion (**d**) and survival in patients with COVID-19 and pneumothorax

Our results demonstrated that the presence of pulmonary bullae (p = 0.005) and pleural effusion (p = 0.003) are associated with lower survival rates in patients with COVID-19 and pneumothorax (Table 4; Fig. 3). However, there was no significant association between survival time and side of pneumothorax (p = 0.252) and mechanical ventilation (p = 0.831).

**Table 4:** Survival analysis and the respiratory and radiological factors

Variable		Mean	95% CI	Log rank P-value
Mechanical ventilation	No	10.16	4.09–16.23	0.831
	Yes	9.93	3.88–15.98	
Side	Left	7.75	3.34–12.16	0.252
	Right	9.00	2.95–15.04	
	Bilateral	17.87	4.41–31.34	
Bullae	No	12.54	7.09–17.98	**0.005**
	Yes	3.44	0.74–6.14	
Pleural effusion	No	2.38	6.57–15.90	**0.003**
	Yes	0.64	0.23–2.76	

### Treatment factors

**Fig. 4:**
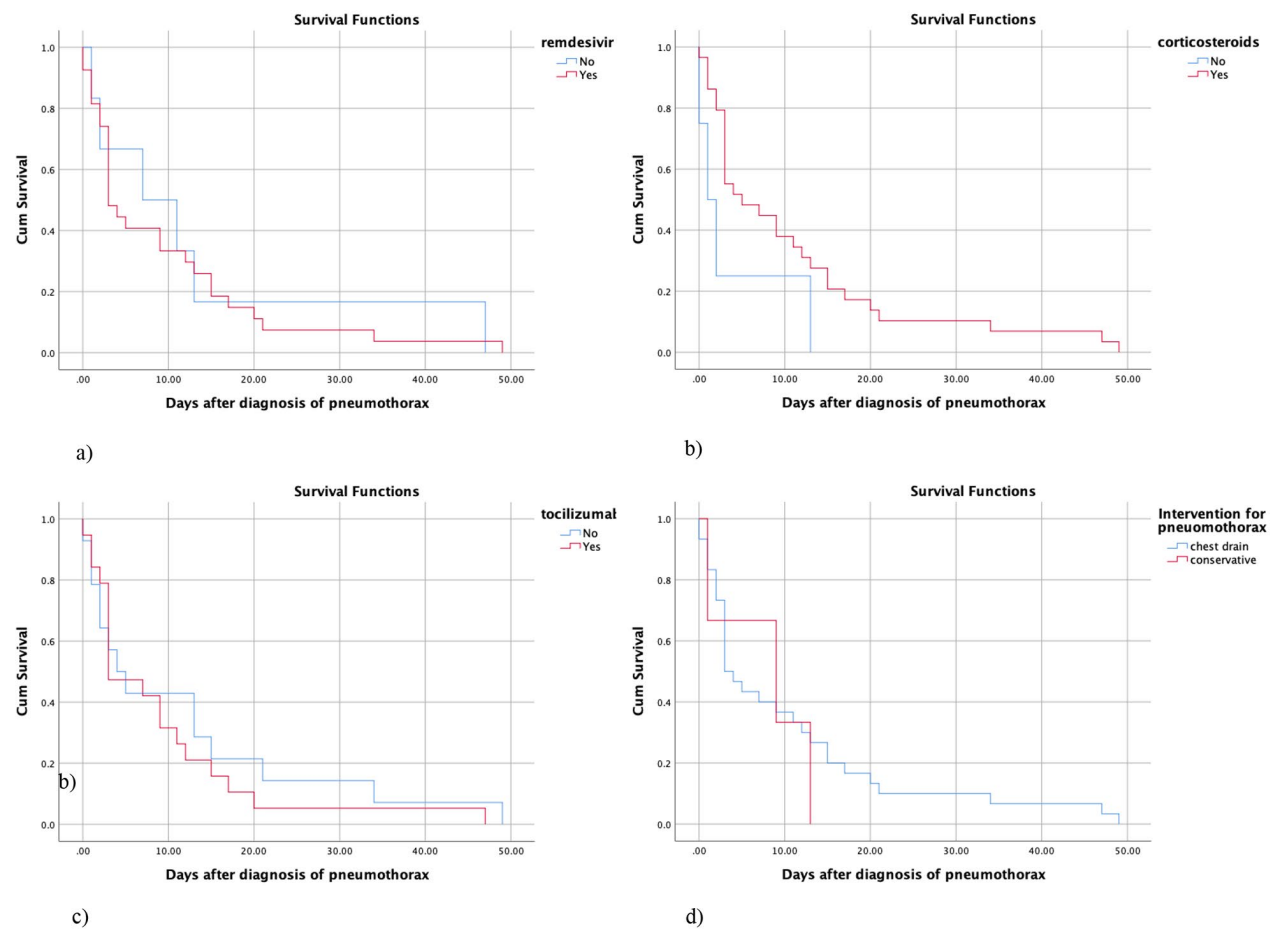
Relationship between treatment with remdesivir (**a**), treatment with corticosteroids (**b**), treatment with tocilizumab (**c**), type of pneumothorax management (**d**) and survival in patients with COVID-19 and pneumothorax

Our results demonstrated no significant association between the treatment factors, including remdesivir (p = 0.790), corticosteroids (p = 0.082), tocilizumab (p = 0.494), and intervention for pneumothorax (p = 0.672) with survival time (Table 5; Fig. 4).

**Table 5:** Survival analysis and the treatment factors

Variable		Mean	95% CI	Log rank P-value
Remdesivir	No	13.50	0-27.71	0.790
	Yes	9.29	4.98–13.60	
Corticosteroids	No	4.00	0-9.93	0.082
	Yes	10.89	6.20-15.59	
Tocilizumab	No	11.63	4.06–19.22	0.494
	Yes	8.89	3.97–13.81	
Intervention	Chest drain	10.30	5.67–14.92	0.672
	Conservative	7.66	0.75–14.58	

## Discussion

This study described a relatively large group of patients with COVID-19 and pneumothorax and investigated the predictors of survival after diagnosis of pneumothorax. A total of 67 patients with COVID-19 and pneumothorax were identified. Of these, 40.7% were located in the left lung, 40.7% were in the right lung, and 18.6% were found bilaterally. Averagely, the pneumothorax occupied 21.73 (15.96) percent of the lung. At the time of pneumothorax diagnosis, the breathing of 39.3% and 25% of patients were with reserved and simple masks, respectively. The most common symptoms in patient with pneumothorax were dyspnea (65.7%), increased cough severity (53.7%), chest pain (25.4%), and hemoptysis (16.4%). The majority of patients had severe (71.6%) and moderate (25.4%) COVID-19. The frequency of pulmonary left and right bullae, pleural effusion, fungal ball was 22.4%, 22.4%, 22.4%, and 7.5%, respectively. The patients were treated with remdesivir (79.1%), corticosteroids (80.6%), and tocilizumab (46.3%). Pneumothorax was managed with chest drain (80.6%), chest drain and surgery (6%), and conservatively (13.4%). The duration of chest tube was 11.60 (10.12) days. The 50-day mortality rate was 52.2% (35 patients) in this period. The average survival time for deceased patients was 10.06 (2.17) days. Our results demonstrated that the presence of pulmonary bullae and pleural effusion is associated with lower survival time in patients with COVID-19 and pneumothorax. However, there was no significant association between survival time and other demographic, clinical, and radiological factors.

The buildup of air between the visceral and parietal pleura, which lines the lungs, is known as a pneumothorax. While a secondary spontaneous pneumothorax is a consequence of underlying lung illness, a primary spontaneous pneumothorax can happen without any triggering event. Although the exact cause of the injury is unknown, the infection-related alveolar damage and an alveolar wall rupture brought on by increased pressure from the intense coughing that occurs in reaction to the virus may be the primary causes [21]. Additionally, during lung infections, an inflammatory response may potentially contribute to secondary spontaneous pneumothorax. Inflammatory exudates may be involved in the development of cysts as a result of severe acute respiratory syndrome, even in the absence of mechanical ventilation, according to certain investigations [22, 23]. Cellular fibromyxoid exudates, which create a valve in the bronchus, can cause pulmonary cystic lesions [24]. Now known as the primary cause of severe acute respiratory syndrome coronavirus 2 infections, cytokine-storm syndrome is a critical clinical condition brought on by a cascade of cytokine activation. It is characterized by overwhelming systemic inflammation, hyperferritinemia, haemodynamic instability, and multiple organ failure [25]. Patients who have encountered respiratory failure during the current COVID-19 pandemic are often exposed to COVID-19 protocols while they are in emergency departments, which may include the potential of positive-pressure ventilation, which might exacerbate the clinical course of a pneumothorax.

Pneumothorax has been noted as a possible, albeit infrequent, consequence of COVID-19 ever since the first instances were published. Out of 99 verified COVID-19 instances with a pneumothorax, Chen et al. [13] identified only one patient with secondary spontaneous pneumothorax. In a study on 92 autopsies, Yang et al. [16], only discovered one case with the same diagnosis. Moreover, pneumothorax was not prevalent, according to an investigation of CT data by Salehi et al. [26].

Pneumothorax can result from COVID-19 pneumonia’s late sequelae [27]. In the research by Ulutas et al. [20], two subjects had been treated with COVID-19 pneumonia about a month previously, released from the hospital, and then later readmitted due to a pneumothorax. Another patient just required a tube thoracostomy, and the patient was discharged three days later after making a full recovery. The authors proposed that in these instances, pneumothorax may be identified as COVID-19 pneumonia late sequela consequence [20].

Pneumothorax during coronavirus infection has been hypothesized to be a significant prognostic factor in the past [28]. However, the treatment of pneumothorax may result in more comorbidities and difficulties. Notably, the insertion of a chest drain to treat a pneumothorax may be regarded as an aerosol-generating technique, and RNA from the severe acute respiratory syndrome coronavirus has recently been found in the pleural fluid at postmortem [29, 30]. When performing aerosol-generating procedures like inserting chest drains, clinicians must be given the proper personal protective equipment. It is also crucial to implement droplet-minimizing modifications, such as digital drainage systems, connecting the drainage circuit to a wall suction line, and using filters to reduce viral spread [31]. It is critical to establish preventative strategies for essential actions after knowing the process of probable COVID-19 transmission during the management of pneumothorax. In our study, the majority were treated with chest drain and no adverse effect was observed. There was no association between the management of pneumothorax (chest drain versus conservative treatment) and the survival of patients. Also, pharmacological treatment of COVID-19 was not associated with survival.

Patients with COVID-19 who have NIV and mechanical ventilation treatment have an increased risk of pneumothorax [32]. Cases of spontaneous pneumothorax identified before NIV and mechanical ventilation were examined by Ulutas et al. [20]. In contrast to other patients, those who required NIV and mechanical ventilation following a pneumothorax had a worse clinical outcome, a longer hospital stay, and increased morbidity and death rates. Three of the five (60%) deaths occurred during ventilation (3 received NIV, 1 received mechanical ventilation and 1 received both). However, we did not show a significant association between mechanical ventilation and decreased survival. Nonetheless, we showed that the presence of bullae or pleural effusion is associated with decreased survival.

In conclusion, this is of the few large-scale studies reporting survival in patients with COVID-19 and pneumothorax. Our results demonstrated that those with pleural effusion or pulmonary bullae have a lower survival time. Further studies are required to investigate the incidence and causality relation between COVID-19 and pneumothorax.

## Data Availability

Input data for the analyses are available from the corresponding author on request.
